# Assessment and treatment of left ventricular pseudoaneurysm due to wire injury in transcatheter aortic valve implantation: a case report

**DOI:** 10.3389/fcvm.2025.1676337

**Published:** 2025-09-22

**Authors:** Fumiko Yoshimachi, Tomohiko Shindo, Shigeo Godo, Kiyotaka Hao, Takashi Shiroto, Jun Takahashi, Satoshi Yasuda

**Affiliations:** Department of Cardiovascular Medicine, Tohoku University Graduate School of Medicine, Sendai, Miyagi, Japan

**Keywords:** left ventricular pseudoaneurysm, aortic valve stenosis, transcatheter aortic valve implantation, wire injury, postoperative complications

## Abstract

Transcatheter aortic valve implantation (TAVI) has recently become a minimally invasive alternative to surgical aortic valve replacement. However, it remains associated with potentially life-threatening complications. Among these, left ventricular pseudoaneurysm is a rare but serious event. Although infrequently reported, its occurrence may be followed by rapid clinical deterioration and fatal outcomes. We report the case of an 84-year-old woman with severe aortic stenosis (AS), who developed a left ventricular pseudoaneurysm following TAVI. Pre-procedural echocardiography showed a heavily calcified and immobile aortic valve, with a peak velocity of 4.0 m/s, an aortic valve area of 1.17 cm², and a mean pressure gradient of 38 mmHg. A self-expandable valve was selected to optimize supra-annular deployment. One day after the procedure, imaging revealed a pseudoaneurysm at the left ventricular apex. This case highlights a rare but critical complication of TAVI. We discuss potential mechanisms, preventive considerations, and management strategies for such events.

## 1 Introduction

The prevalence of aortic stenosis (AS) has been steadily rising with the aging of the population, particularly among elderly individuals (1–3). Transcatheter aortic valve implantation (TAVI) has emerged as a less invasive alternative to surgical aortic valve replacement and is increasingly performed in this population due to its favorable recovery profile. Despite its advantages, TAVI is associated with a range of serious complications (4, 5). Among these, left ventricular pseudoaneurysm (LVP) is an rare but potentially fatal complication. Its infrequent occurrence and often nonspecific clinical presentation may delay recognition and diagnosis (6, 7). This case report demonstrates an occurrence of LVP following TAVI, with an atypical clinical course. We also discuss the clinical context in which this complication should be considered and explore potential mechanisms underlying its development.

An 84-year-old woman with a long-standing history of heart murmur and progressive dyspnea was referred for the evaluation and management of severe AS. A decade earlier, transthoracic echocardiography had revealed a left ventricular–aortic (LV-Ao) mean pressure gradient of 17 mmHg. At the time of referral, this had progressed to 51 mmHg, indicating hemodynamically severe AS. Her medical history included diabetes mellitus and dyslipidemia, both under pharmacological control.

Transthoracic echocardiography demonstrated a preserved left ventricular ejection fraction of 80%, hyperdynamic left ventricular contraction, mildly accelerated intraventricular flow, mitral annular calcification, and a small pericardial effusion measuring 1.5 mm. Since there was no history of collagen disease or pericarditis, the etiology of the pericardial effusion was unclear. A computed tomography (CT) scan from another hospital showed pericardial effusion having been present for the past five years, and the amount had not increased over time. This CT also showed low Right Coronary Artery (RCA) height and small Sinus of Valsalva (SOV), indicating a relatively high risk of coronary artery occlusion. However, there were no obvious coronary artery stenosis was found, and there were no findings suggestive of a previous myocardial infarction.

**Figure 1:**
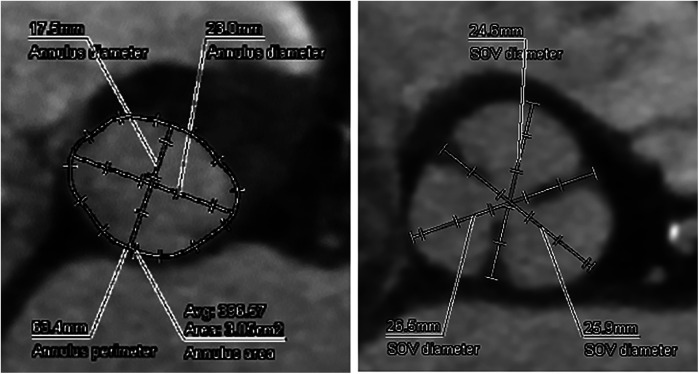
Preoperative CT analysis of the A-valve complex. Annulus perimeter was 63.4 mm, annulus area was 305 mm^2^, suggesting a narrow annulus.

The aortic valve was heavily calcified with restricted mobility, a peak transvalvular velocity of 4.0 m/s, an aortic valve area of 1.17 cm², and a mean pressure gradient of 38 mmHg. Preprocedural thoracoabdominal CT showed marked aortic valve calcification and an aortic annulus with a perimeter of 63.0 mm and an area of 304 mm² (Figure 1). Although the left ventricular contraction was excessive and left ventricular wall thickness had concentric remodeling, there was no evidence of asymmetric septal hypertrophy, systolic anterior movement, or LVOT stenosis.

## 2 Case report

### 2.1 Case presentation

Following multidisciplinary discussion with the heart team, transcatheter aortic valve implantation (TAVI) was selected as the treatment strategy. The perioperative risk was estimated at 1.23% using the European System for Cardiac Operative Risk Evaluation (EuroSCORE II) and 3.59% using the Society of Thoracic Surgeons (STS) score. Aortic valves with an annulus diameter ≤21 mm are generally considered narrow, and the SMART trial defined a narrow annulus as one with an area ≤430 mm² (8). Given the small annular area in this case, a self-expanding prosthetic valve was chosen to facilitate supra-annular deployment and optimize hemodynamic performance.

**Figure 2:**
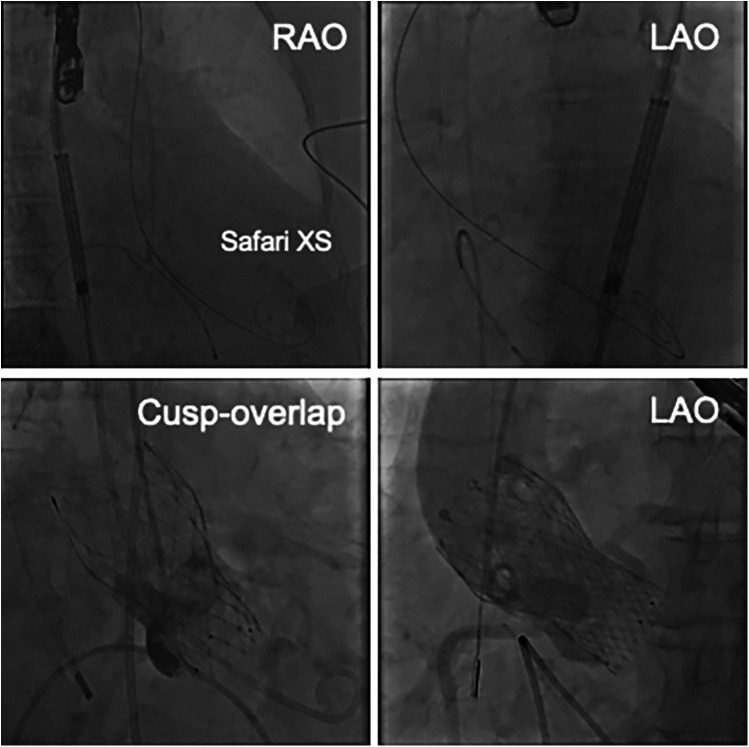
The upper images show the position of the Safari XS in the LV. The lower images show the cusp-overlapping view and the LAO view after valve placement.

After vascular access was obtained via the left femoral artery, a DrySeal sheath was inserted. The aortic valve was initially crossed using an Amplatz Left (AL-1) catheter and a straight guidewire. This was then exchanged for a stiff angle guidewire, which was positioned in the LV apex to provide stable support for the delivery system. Under fluoroscopic and intracardiac echocardiographic (ICE) guidance, the self-expanding prosthetic valve was advanced across the native aortic valve. During deployment, partial recapture and repositioning were required to achieve optimal alignment. The valve was ultimately implanted with the lower end positioned to avoid direct contact with the membranous septum. Final deployment depths were 3 mm below the annulus on both the non-coronary cusp (NCC) and left coronary cusp (LCC) sides (Figure 2). Given the preoperative CT findings of moderate pericardial effusion, a small thoracotomy was performed after TAVI, and 400 ml of pericardial fluid was drained by surgical thoracotomy, incision from the xiphoid process. Post-procedural transthoracic echocardiography revealed no wall motion abnormalities, no residual pericardial effusion, and no abnormalities at the LV apex. At follow-up, transthoracic echocardiography demonstrated stable valve function with a peak transvalvular velocity of 1.95 m/s, an aortic valve area of 1.80 cm², and a mean pressure gradient of 8 mmHg. Mild paravalvular aortic regurgitation was observed at two sites.

**Figure 3:**
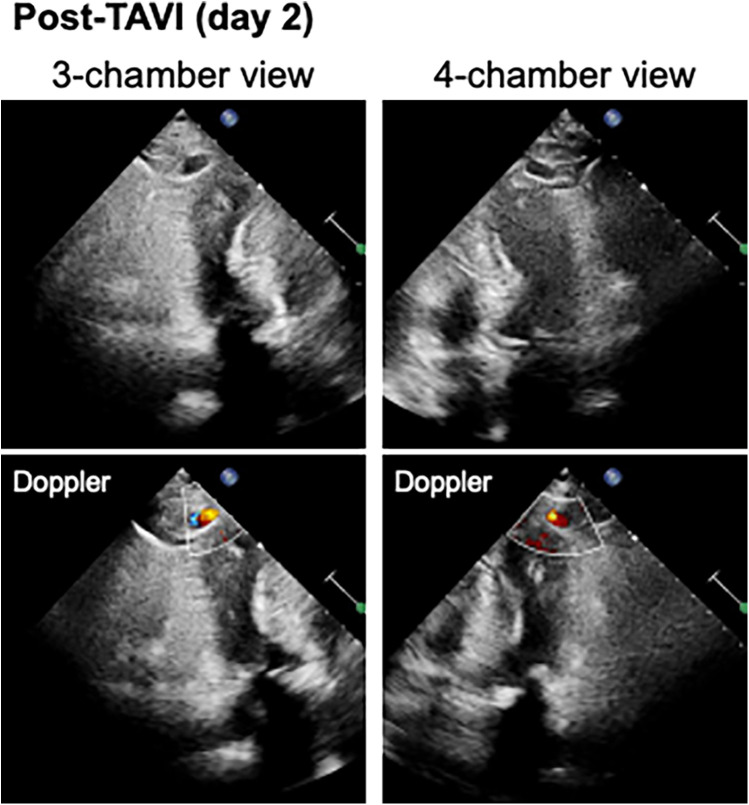
The color doppler echocardiography visualized a cavity and abnormal blood flow inflow within the apical structures consistent with a pseudoaneurysm.

**Figure 4:**
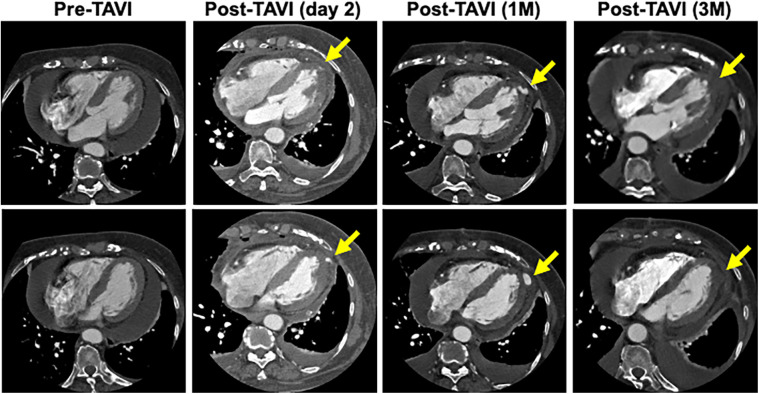
Time course of the morphology of the pseudoaneurysm. CT scan taken 3 months after repair surgery showed that the lumen of the pseudoaneurysm had disappeared.

A blood test obtained one day after TAVI revealed marked elevation of myocardial enzymes: creatine kinase (CK) at 1921 U/L and CK-MB at 236 U/L. Despite these findings, the patient did not report chest pain, and electrocardiography showed no ischemic changes such as ST-segment elevation or new Q waves. Given the absence of clinical and electrocardiographic evidence of acute myocardial infarction, alternative causes of enzyme elevation were considered. These included myocardial injury secondary to mechanical stress from the procedure itself, microvascular embolization, temporary guidewire-induced trauma, or global ischemia due to rapid pacing during valve deployment. Initial transthoracic echocardiography revealed no regional wall motion abnormalities. However, a repeat study performed three hours later identified new, localized hypokinesis at the left ventricular apex, although the volume of pericardial effusion remained unchanged. To further investigate, contrast-enhanced CT was performed five hours after the enzyme elevation was noted. This allowed us to confirm the presence of a pseudoaneurysm at the left ventricular apex. And, by color doppler echocardiography, we also confirmed abnormal flow within the newly visualized apical structure, consistent with a pseudoaneurysm that was seen in the CT scanning (Figure 3). Due to the findings of rapidly expanding of the pseudoaneurysm, and the extremely high potential risk of cardiac rupture, emergency surgical intervention was undertaken. Intraoperatively, a pseudoaneurysm measuring approximately 7 mm was identified on the lateral aspect of the left ventricular apex. The surrounding myocardial tissue was noted to be fragile, suggesting localized necrosis or structural compromise. Surgical repair was performed using two bovine pericardial patches (4 × 1 cm), which were sutured in place with continuous stitching. Postoperatively, the pseudoaneurysm cavity was largely thrombosed, and follow-up CT imaging demonstrated a reduction in the contrast-enhancing area to approximately 5 mm (Figure 4). One month later, repeat CT imaging showed no residual aneurysmal structure. She was transferred from the intensive care unit on postoperative day 9 and discharged from the hospital on day 27. She continues to do well on outpatient follow-up.

### 2.2 Discussion

We report a case of LVP likely caused by wire-related injury during TAVI. While most previously reported cases of TAVI-related LVP have involved the transapical approach (6, 7), this case highlights the potential for such complications to occur even with the transfemoral approach. We discuss possible procedural factors that may contribute to LVP formation in transfemoral TAVI and how they can be addressed. Although ventricular perforation is often attributed to the use of a straight wire during initial passage through the left ventricle (9, 10), pseudoaneurysm formation related to angled wires has been rarely reported.

Left ventricular pseudoaneurysms are typically identified during or immediately after TAVI. In this case, however, the pseudoaneurysm was detected the day after the procedure and demonstrated rapid expansion within a few hours of its initial recognition (7). A commonly described mechanism involves perforation of the left ventricular wall by a straight-tipped wire used to cross the aortic valve, creating a myocardial breach. In the present case, however, fluoroscopic imaging did not suggest that the straight wire had contacted the ventricular wall. Unlike straight wires, angled wires are generally considered less traumatic due to their softer, curved tips. However, in this case, the possibility remains that even a minor injury caused during wire manipulation may have contributed to pseudoaneurysm formation, particularly under high left ventricular systolic pressure. This may help explain the delayed appearance and subsequent rapid expansion of the pseudoaneurysm. The post-procedural elevation of myocardial enzymes could reflect myocardial injury related to this process, although other causes cannot be ruled out. While injury from the straight wire cannot be definitively excluded, the fluoroscopic images showing the position and direction of the angled wire suggest it as a plausible source of myocardial injury in this case. In addition, we performed multiple recaptures due to difficulties in placing the prosthetic valve at the appropriate depth, which may have placed stress on the left ventricle and contributed to the development of a pseudoaneurysm. Also, CT scans revealed an increase in pericardial fluid over time, but the CT values suggested that this was due to a postoperative reaction rather than bloody pericardial fluid.

To prevent similar complications, several procedural considerations are essential. First, even with angled wires, careful manipulation is critical within the left ventricle. Operators should avoid exerting force when advancing or withdrawing the wire in constrained positions, particularly when the guiding catheter (e.g., AL-1) is positioned near the ventricular wall. Partial withdrawal of the angled wire into the left ventricular cavity before disengaging the guiding catheter may help reduce the risk of myocardial contact. Second, close clinical and imaging follow-up is warranted in the perioperative period. Complications such as pseudoaneurysm or infection may not manifest immediately after TAVI, and early detection is key to prompt intervention and improved outcomes.

Treatment options for LVP include medical management, percutaneous interventions, and surgical repair, depending on the size, location, and clinical course. Conservative follow-up may be considered for stable pseudoaneurysms measuring smaller than 3.0 cm in diameter (11). For lesions exceeding 3.0 cm or demonstrating progressive enlargement, intervention is generally recommended. In the present case, surgical repair was chosen due to the rapid expansion of the pseudoaneurysm and concern for impending rupture.

In conclusion, LVP can occur during transfemoral TAVI, possibly from wire injury. Careful wire handling and monitoring of CK/CK-MB post-procedure are important to facilitate early detection and prompt management of this rare complication.

## Data Availability

The original contributions presented in the study are included in the article/Supplementary Material, further inquiries can be directed to the corresponding author.

## References

[1] Ambrosy AP Go AS Leong TK Garcia EA Chang AJ Slade JJ Temporal trends in the prevalence and severity of aortic stenosis within a contemporary and diverse community-based cohort. Int J Cardiol. (2023) 384:107–11. 10.1016/j.ijcard.2023.04.047 37119944

[2] Osnabrugge RL Mylotte D Head SJ Van Mieghem NM Nkomo VT LeReun CM Aortic stenosis in the elderly: disease prevalence and number of candidates for transcatheter aortic valve replacement: a meta-analysis and modeling study. J AM Coll Cardiol. (2013) 62(11):1002–12. 10.1016/j.jacc.2013.05.015 23727214

[3] Martinsson A Li X Andersson C Nilsson J Smith JG Sundquist K. Temporal trends in the incidence and prognosis of aortic stenosis: a nationwide study of the Swedish population. Circulation. (2015) 131(11):988–94. 10.1161/CIRCULATIONAHA.114.012906 25779541

[4] Laborde JC Brecker SJ Roy D Jahangiri M. Complications at the time of transcatheter aortic valve implantation. Methodist Debakey Cardiovasc J. (2012) 8(2):38–41. 10.14797/mdcj-8-2-38 22891127 PMC3405794

[5] Arnold SV Zhang Y Baron SJ McAndrew TC Alu MC Kodali SK Impact of short-term complications on mortality and quality of life after transcatheter aortic valve replacement. JACC Cardiovasc Interv. (2019) 12(4):362–9. 10.1016/j.jcin.2018.11.008 30784641 PMC6392020

[6] Vanezis AP Baig MK Mitchel IM Shajar M Naik SK Henderson RA Pseudoaneurysm of the left ventricle following apical approach TAVI. J Cardiovasc Magn Reson. (2011) 13(1):79. 10.1186/1532-429X-13-79 22152296 PMC3253049

[7] Morjan M El-Essawi A Anssar M Harringer W. Left ventricular pseudoaneurysm following transfemoral aortic valve implantation. J Card Surg. (2013) 28:510–1. 10.1111/jocs.1214 23866003

[8] Herrmann H Mehran R Blackman D Baliey S Mollmann H Abdel-Wanab M Self-expanding or balloon-expandable TAVR in patients with a small aortic annulus. N Eng J Med. (2024) 390:1959–71. 10.1056/NEJMoa2312573 38587261

[9] Ichise T Nagata Y Nakatsuji H Fukagawa H Kashiyama N Taniguchi K Guidewire-induced left ventricular perforation related to thoracic aorta tortuosity during transcatheter aortic valve implantation. J Transcatheter Valve Ther. (2024) 6(1):1–4. 10.33290/jtvt.cr.23-0005

[10] Abe R Higuchi R Shimizu A Saji M Takamisawa I. A pre-shaped guidewire could cause left ventricular perforation during transcatheter aortic valve implantation: pre-shaped guidewire LV perforation in TAVI. AsiaIntervention. (2021) 7(2):112–3. 10.4244/AIJ-D-21-00025 34913015 PMC8658554

[11] Torchio F Garatti A Ronco D Matteucci M Massimi G Lorusso R. Left ventricular pseudoneurysm: the niche of post-infaction mechanical complications. Ann Cardiothorac Surg. (2022) 11(3):290–8. 10.21037/acs-2022-ami-25 35733717 PMC9207692

